# Effectiveness of a culturally adapted psychoeducational intervention for family caregivers of children with hematologic malignancy: a randomized controlled trial

**DOI:** 10.3389/fpsyt.2026.1835671

**Published:** 2026-06-02

**Authors:** Zhiyu Ye, Yuanyuan Zhang, Lisha Dai, Wentian Li, Fang Wang

**Affiliations:** 1 Key Laboratory of Adolescent Cyberpsychology and Behavior (CCNU), Ministry of Education, School of Psychology, Central China Normal University, Wuhan, China; 2 Department of Pediatrics, Union Hospital,Tongji Medical College, Huazhong University of Science and Technology, Wuhan, China; 3 Department of Psychiatry, Wuhan Mental Health Center, Department of Psychosomatic, Wuhan Hospital for Psychotherapy, Research Center for Psychological and Health Sciences, Wuhan, China; 4 Department of Psychosomatic Medicine, Shanghai East Hospital, Tongji University School of Medicine, Shanghai, China

**Keywords:** cultural adaptation, family caregivers, pediatric hematologic malignancy patients, psychoeducation intervention, randomized controlled trial

## Abstract

**Objective:**

This study aimed to develop and evaluate a culturally adapted psychoeducational intervention to address the severe psychological distress and caregiving burden commonly experienced by caregivers of newly diagnosed pediatric hematologic malignancy patients during the early stages of illness.

**Methods:**

Among 116 randomized caregivers, 90 completed all three assessments and were included in the complete-case analysis. The intervention group received a 4-week psychoeducation program delivered by medical social workers, whereas the control group received routine care. Outcomes were assessed at baseline, post-intervention, and 48-day follow-up.

**Results:**

Compared with controls, caregivers in the intervention group showed greater reductions in depressive and post-traumatic stress symptoms after the intervention, and these reductions were maintained at follow-up. Caregiver burden showed a delayed reduction, with between-group differences becoming apparent at follow-up. Anxiety levels decreased over time in both groups, but the group × time interaction was not statistically significant.

**Conclusion:**

The culturally adapted psychoeducational intervention was associated with reductions in depressive and post-traumatic stress symptoms and a delayed reduction in caregiver burden, but did not show an additional effect on anxiety beyond routine care. These findings provide preliminary support for structured, culturally responsive psychoeducational support in pediatric hematology settings.

## Introduction

Hematologic malignancy (HM) refers to malignant tumors originating from the hematopoietic and lymphatic systems, characterized by abnormal proliferation and differentiation of blood cells. It primarily includes various types of leukemia, multiple myeloma, and malignant lymphoma. Among malignant tumors in children and adolescents, hematologic malignancies are among the most common, with leukemia being a major component of pediatric cancer burden (1). The disease burden in China is substantial: according to data from the National Cancer Center, the age-standardized incidence rate of childhood malignancies is 87.1 per million and has continued to increase, with the highest incidence observed in the 0–4 age group (2). These diseases typically involve prolonged and complex treatment, repeated hospitalization, and high care demands, imposing substantial physical and psychological burdens on pediatric patients while profoundly affecting family caregivers.

Family caregivers, most commonly parents, are central to the care of children with hematologic malignancy. During the early diagnostic and treatment phase, they must rapidly adapt to the child’s illness, communicate with healthcare professionals, manage treatment-related information, provide daily and emotional care for the child, and reorganize family roles. This period is therefore not only a medical transition but also a major psychosocial stressor for the family. Studies of caregivers of children newly diagnosed with leukemia, lymphoma, and other pediatric cancers have shown elevated emotional symptoms, including anxiety, depression, and distress, particularly around diagnosis and early treatment, although symptom trajectories may change over time (3). A claims-based cohort study also found higher mental health care utilization among parents of children with cancer than among parents of children without cancer (4). Together, these findings support the need for timely psychosocial support for caregivers during the newly diagnosed phase of pediatric cancer care (5).

Psychoeducation is one commonly recommended form of psychosocial support for families affected by pediatric cancer (6). Pediatric psychosocial care standards recommend that children with cancer and their family members receive developmentally appropriate psychoeducation, information, and anticipatory guidance about disease, treatment, procedures, coping, and psychosocial adjustment (7). Psychoeducational interventions typically combine illness-related information, anticipatory guidance, coping skills, problem-solving strategies, emotional support, and guidance on accessing available resources. Evidence from randomized trials and meta-analytic work among caregivers of children with cancer suggests that psychoeducational interventions may improve post-traumatic stress symptoms, mood, and problem-solving skills, although effects are not consistently superior to usual care for all outcomes, including anxiety and depression (8). Similarly, problem-solving skills training for mothers of children newly diagnosed with cancer has shown beneficial effects on maternal adjustment and negative affectivity (9). These findings suggest that psychoeducation may be clinically useful, but its content, delivery format, and target mechanisms need to be closely aligned with caregivers’ actual needs and clinical context (8, 10).

Many psychoeducational interventions for cancer caregivers have been developed and evaluated outside the Chinese pediatric oncology context, and their assumptions about communication, emotional expression, support seeking, and caregiver autonomy may not fully correspond to caregiving practices in Chinese families (11). In China, caregiving is often embedded in strong family responsibility, family-based decision-making, and reliance on both medical authority and informal family or acquaintance networks (12). These features may become particularly salient in pediatric cancer care, where parents must simultaneously manage treatment-related information, protect the child emotionally, support treatment adherence, and coordinate family resources (13). As a result, caregivers may face culturally and clinically specific dilemmas, such as how to communicate with the child about diagnosis and treatment, how to respond to the child’s fear or treatment resistance, how to balance protection and disclosure, and how to mobilize family and social support (14). Therefore, directly transferring psychoeducational content developed in other sociocultural contexts may be insufficient without adaptation to local caregiving practices and communication dilemmas (11). Cross-cultural intervention research similarly emphasizes that cultural adaptation should go beyond linguistic translation and should align intervention content and delivery with local values, social structures, and care practices (15). Consistent with this perspective, formative qualitative work with local caregivers was used before the trial to identify key caregiving concerns to be addressed in a culturally adapted psychoeducational intervention, particularly illness-related communication, treatment adaptation, caregiver emotional regulation, and family/social support. These locally identified concerns shaped the intervention manual used in the present trial.

Based on this rationale and formative work, the present study developed and evaluated a manual-guided psychoeducational intervention for family caregivers of children newly diagnosed with hematologic malignancy in China. We examined whether caregivers receiving the intervention, compared with those receiving routine care, showed greater improvement in depressive symptoms, anxiety symptoms, post-traumatic stress symptoms, and caregiver burden from baseline to post-intervention and short-term follow-up. By integrating caregiver needs identified in the local clinical context with structured psychoeducational content, this study aimed to provide preliminary evidence for a feasible caregiver-focused psychosocial support model in pediatric hematology settings.

## Methods

This study was a single-blind, parallel-group randomized controlled trial using a single-factor repeated-measures design. The independent variable was the psychoeducational intervention condition (intervention group vs. control group), and the dependent variables were mental health outcomes, including depressive symptoms, anxiety symptoms, post-traumatic stress symptoms, and caregiver burden. All outcomes were assessed at three time points: baseline (T0), post-intervention (T1), and follow-up (T2).

### Participants

Participants were recruited from the Pediatric Hematology Department of Union Hospital, Tongji Medical College, Huazhong University of Science and Technology. Inclusion criteria: (1) Primary family caregiver (e.g., parent) of a newly diagnosed pediatric hematologic malignancy patient aged 0–18 years; (2) Patient undergoing initial standard treatment; (3) Neither caregiver nor patient had a history of significant physical or mental illness; (4) Ability to adhere to intervention protocols; (5) Only one hematologic malignancy patient in the household; (6) Proficiency in Chinese reading and writing, ability to comprehend study content and sign informed consent; (7) Not concurrently participating in other similar studies. Exclusion criteria: (1) Ceasing to be the primary caregiver during the study period; (2) Child receiving palliative care; (3) Child’s death during the study period. Withdrawal criteria: (1) Caregiver voluntarily requests to withdraw from the intervention; (2) Refusal to undergo relevant measurement assessments.

### Sample size

An *a priori* power analysis was conducted using GPower 3.1. To detect a medium effect size (Cohen’s d = 0.6) with 80% power at a two-sided alpha level of 0.05 and an allocation ratio of 1:1, a minimum of 45 participants per group was required. Anticipating a 20% attrition rate, we aimed to recruit 57 participants per group, totaling 114 participants.

### Recruitment and randomization

The recruitment period for this study spanned from June 1, 2023, to June 30, 2025. Potential eligible participants were identified with the assistance of ward nurses and medical social workers. An independent researcher, not involved in other study processes, obtained their written informed consent and randomly assigned them to either the experimental or control group in a 1:1 ratio. Participants in the experimental and control groups were placed in two separate wards within the same department to minimize contact and information contamination between groups. Regarding blinding, due to the nature of the intervention, blinding of participants and intervention providers was not feasible. However, outcome assessors and data analysts were strictly blinded.

### Materials

The core intervention material was the self-developed “Psychological Education Manual for Caregivers of Pediatric Hematologic/Oncologic Patients”. This manual was systematically developed based on formative qualitative work conducted before the trial: semi-structured interviews with 12 target caregivers and grounded theory analysis identified their psychological experiences and core needs during the initial diagnosis phase. The manual’s content closely addresses these psychosocial needs while integrating stress theory, cognitive development theory, and social support theory as a guiding framework, supporting the intervention’s theoretical grounding and cultural relevance. The main body of the manual is divided into two sections, providing structured psychoeducation:(1) Child Psychological Adjustment: Focuses on disease-related communication with the child, helping them adapt to treatment and hospital/home life, and provides differentiated communication guidelines based on cognitive development stages; (2) Caregiver Psychological Adjustment: Concentrates on caregivers’ emotional awareness and management, stress relief techniques, accessing social support resources, and balancing the needs of other family members. The manual adopts a problem-solving approach, incorporating specific scenario strategies and practical examples.

### Intervention

The intervention was delivered as a manual-guided, semi-structured psychoeducational program rather than as a verbatim scripted intervention. Two trained medical social workers delivered the intervention individually and face-to-face. The psychoeducational manual served as the core delivery guide and specified the main modules, key messages, suggested communication examples, and practical problem-solving strategies to be covered in each session. At the same time, social workers were allowed to adjust the order and emphasis of the content according to caregivers’ immediate concerns, while ensuring that all core modules were addressed.

The intervention consisted of two main sessions and one brief review session. The first session, conducted approximately during the second week after enrollment, focused on the child’s psychological adjustment, including disease-related communication with the child, helping the child adapt to treatment, and supporting the child’s hospital and home adjustment. The second session, conducted during weeks 3–4, focused on the caregiver’s psychological adjustment, including emotional awareness and regulation, stress coping, use of social support resources, and balancing the needs of other family members. Each session lasted approximately 20 minutes. During week 4, a brief review and Q&A session of approximately 15 minutes was conducted to consolidate the content and respond to caregivers’ questions. After each session, social workers documented session completion and key content delivered, and caregivers completed a short feedback form to assess their understanding of the psychoeducational content.

Caregivers in both groups received the same routine ward-based support after admission. This included standard medical care, disease-related education from healthcare providers, daily care guidance, and routine support from medical social workers, such as consultation regarding financial assistance resources and general psychosocial support when needed. These services were part of usual care in the ward and were provided irrespective of group allocation. The key difference between groups was that caregivers in the intervention group additionally received the manual-guided structured psychoeducational intervention developed for this study, whereas caregivers in the control group did not receive the psychoeducational manual or the structured psychoeducational sessions.

### Procedures

**Figure 1:**
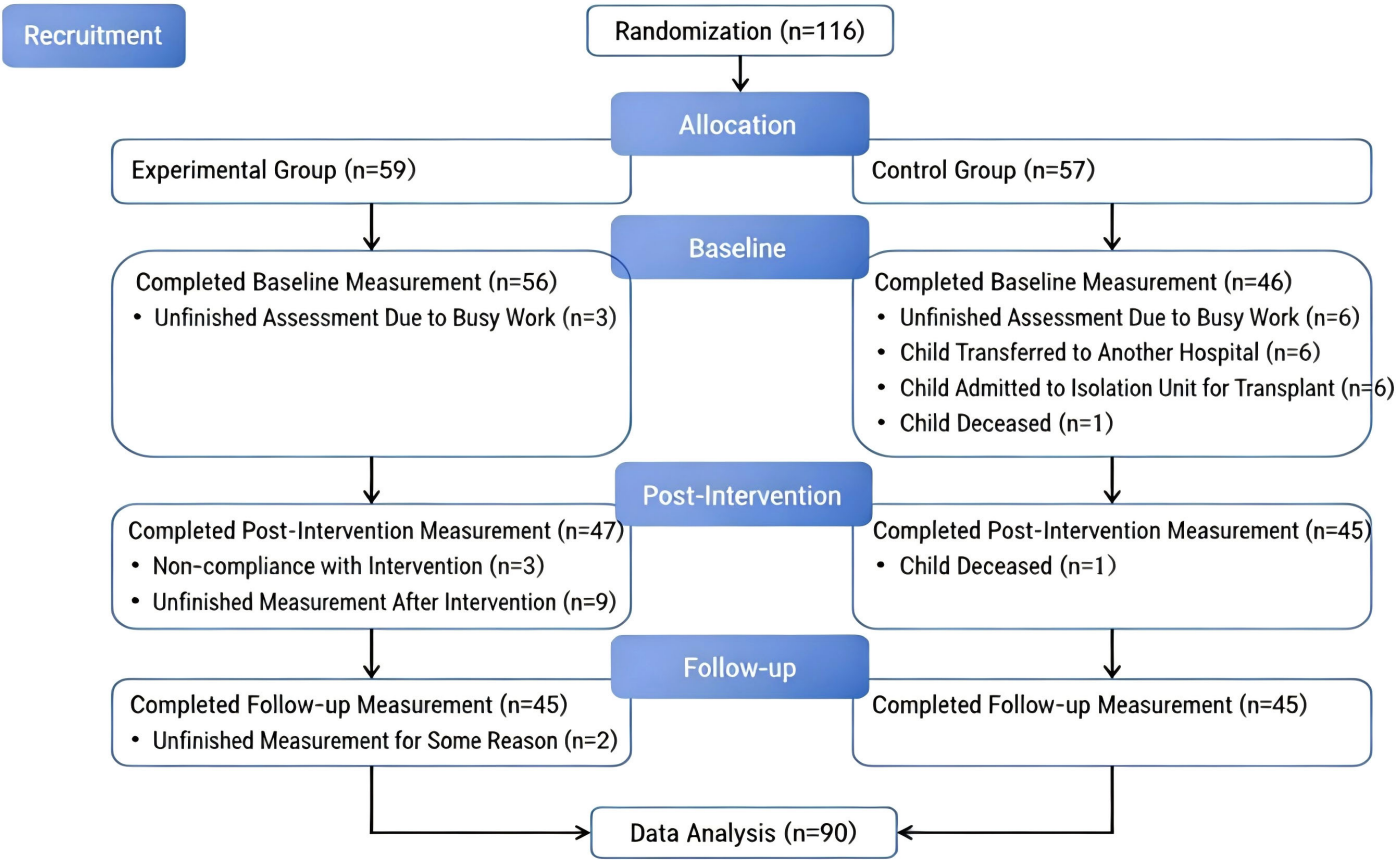
Consort flowchart.

Research assistants, assisted by ward nurses, identified and approached eligible family caregivers. After obtaining written informed consent, participants were randomly assigned to either the experimental or control group. To assess intervention effectiveness, all participants completed questionnaire assessments at three time points. Considering the busy schedule of newly diagnosed caregivers during their first week of hospitalization, the baseline assessment (T0) was uniformly scheduled for the second week after enrollment. Subsequently, participants in the experimental group received the aforementioned approximately two-week individualized psychoeducation intervention (flexibly scheduled for completion within 2–4 weeks). At week 4 post-enrollment (T1), post-intervention assessments were conducted simultaneously for both groups. Finally, at day 48 post-enrollment (T2), follow-up assessments were performed for both groups during the children’s scheduled return to the hospital for routine clinical risk evaluations. All assessments were completed online using electronic devices under the on-site guidance of uniformly trained researchers. The participant enrollment and withdrawal process is illustrated in **Figure 1**.

### Outcome measures

Primary outcome measures were assessed at baseline (T0), post-intervention (T1), and follow-up (T2) using validated Chinese-language self-report scales. Internal consistency was evaluated using Cronbach’s alpha coefficients calculated from the baseline data in the present complete-case analytic sample.

Depressive symptoms. Depressive symptoms were measured using the 9-item Patient Health Questionnaire (PHQ-9). Each item is rated from 0 (“not at all”) to 3 (“nearly every day”), yielding a total score ranging from 0 to 27, with higher scores indicating more severe depressive symptoms. Standard severity categories are 0–4 for minimal or no depression, 5–9 for mild depression, 10–14 for moderate depression, 15–19 for moderately severe depression, and 20–27 for severe depression. The PHQ-9 has been validated in Chinese populations and has shown good reliability and validity (16). In the present complete-case sample, Cronbach’s alpha was 0.950.

Anxiety symptoms. Anxiety symptoms were assessed using the 7-item Generalized Anxiety Disorder scale (GAD-7). Each item is rated from 0 (“not at all”) to 3 (“nearly every day”), producing a total score ranging from 0 to 21, with higher scores indicating more severe anxiety symptoms. Standard severity categories are 0–4 for minimal anxiety, 5–9 for mild anxiety, 10–14 for moderate anxiety, and 15–21 for severe anxiety. The GAD-7 has also been psychometrically evaluated in Chinese samples, with evidence supporting its reliability and validity as a measure of anxiety symptoms (17). In the present complete-case sample, Cronbach’s alpha was 0.956.

Post-traumatic stress symptoms. Post-traumatic stress symptoms were measured using the Post-traumatic Stress Disorder Checklist-Civilian Version (PCL-C). The PCL-C contains 17 items rated on a 5-point scale, with total scores ranging from 17 to 85. Higher scores indicate more severe post-traumatic stress symptoms, and a total score of 38 or above was used to indicate clinically significant post-traumatic stress symptoms in this study. Evidence from Chinese validation research supports the reliability and validity of the PCL-C for assessing post-traumatic stress symptoms (18). In the present complete-case sample, Cronbach’s alpha was 0.975.

Caregiver burden. Caregiver burden was assessed using the Zarit Burden Interview (ZBI). The ZBI contains 22 items rated from 0 to 4, yielding a total score ranging from 0 to 88, with higher scores indicating greater perceived caregiver burden. Scores of 0–20 indicate little or no burden, 21–40 mild burden, 41–60 moderate burden, and 61–88 severe burden. The Chinese ZBI has been widely used in caregiver research and has shown acceptable psychometric properties in Chinese caregiver samples (19). In the present complete-case sample, Cronbach’s alpha was 0.867.

### Quality control

To ensure methodological rigor, multiple quality control measures were implemented. First, allocation concealment was maintained using sealed envelopes based on the random allocation sequence. Second, outcome assessors and data analysts were blinded to group allocation. Third, intervention fidelity was supported through standardized training, a simulated practice session before formal implementation, use of the psychoeducational manual as a delivery guide, documentation of session completion and key content after each session, and caregiver feedback forms assessing comprehension of the delivered content. Fourth, all data were collected through an encrypted electronic data collection platform with on-site guidance from uniformly trained researchers, and standardized procedures were used for data checking and handling outliers.

### Statistical analysis

All data analyses were performed using SPSS 22.0 software, with the significance level set at α = 0.05 (two-tailed). Analyses were conducted using the complete-case sample, defined as participants who completed all three assessments at baseline (T0), post-intervention (T1), and follow-up (T2). Because the primary analysis used repeated-measures ANOVA, participants with missing outcome data at any of the three measurement points were not included in the main analysis. Descriptive statistics, including frequencies, percentages, means, and standard deviations, were used to summarize demographic characteristics and baseline outcome scores for the complete-case analytic sample. Baseline differences between the intervention and control groups were examined using independent-samples t-tests for continuous variables and chi-square tests for categorical variables.

The intervention effect on primary outcome measures (PHQ-9, GAD-7, PCL-C, ZBI) was assessed using repeated measures analysis of variance (ANOVA). For each measure, a 2 (group: experimental vs. control) × 3 (time: baseline T0, post-intervention T1, follow-up T2) repeated measures ANOVA was conducted. First, Mauchly’s sphericity test was performed. If sphericity was violated (*p* < 0.05), Greenhouse-Geisser correction was applied to adjust degrees of freedom and p-values.

The core analysis focused on testing the significance of the “group × time” interaction effect to determine whether intervention effects exhibited distinct temporal patterns across groups. If the interaction effect was significant, simple effects analysis and *post-hoc* comparisons were further conducted. Specifically: (1) Simple effects were tested between groups at each of the three time points; (2) Changes in scores within each group across different time points were tested. All pairwise *post-hoc* comparisons were conducted using the Least Significant Difference (LSD) method, with Bonferroni correction applied to p-values to control Type I errors. For outcome variables showing no significant interaction effect, the main effects of group and time were reported, along with main effect tests and the overall explanatory power of the model (R²). Additionally, participation rates, intervention completion rates, and follow-up dropout rates were calculated to assess the feasibility of the study process.

### Ethics

This study protocol was approved by the Ethics Committees of Wuhan Union Hospital and Wuhan Mental Health Center (No.: KY2023.0606.01). All participants provided written informed consent and received appropriate financial compensation. The study was conducted in accordance with the Declaration of Helsinki. This trial is retrospectively registered on OSF (https://osf.io/wh8vg).

## Results

### Baseline characteristics of the participants

Among the 116 randomized caregivers, 92 (79.3%) completed the post-intervention assessment (T1), and 90 (77.6%) completed the follow-up assessment (T2). The main analyses were based on the complete-case analytic sample of 90 caregivers who completed all three assessments at T0, T1, and T2, including 45 caregivers in the intervention group and 45 in the control group. Participants in the complete-case sample had a mean age of 36.20 ± 5.60 years, and 81.1% were female. No statistically significant differences were observed between the two groups in demographic characteristics (all p > 0.05), indicating acceptable baseline comparability within the complete-case sample.

Baseline assessments in the complete-case sample revealed substantial psychological distress among caregivers: mean scores for depression (PHQ-9: 21.94 ± 7.14) and anxiety (GAD-7: 18.87 ± 6.49) were in the severe range; 62.2% (56/90) exhibited significant post-traumatic stress symptoms (PCL-C ≥ 38); and caregiver burden was moderate to severe (ZBI: 55.86 ± 13.80). No statistically significant between-group differences were observed in baseline outcome scores (**Table 1**). However, baseline anxiety scores were numerically higher in the control group than in the intervention group and approached statistical significance (*p* = 0.055). This numerical baseline imbalance should therefore be considered when interpreting the anxiety-related findings.

**Table 1:** Baseline outcome scores by group in the complete-case analytic sample.

Outcome measure	Intervention group (n=45) M ± SD	Control group (n=45) M ± SD	T (*df* = 88)	*p*
PHQ-9	21.02 ± 6.90	22.87 ± 7.33	-1.229	0.222
GAD-7	17.56 ± 5.74	20.18 ± 6.98	-1.946	0.055
PCL	41.67 ± 19.53	47.58 ± 15.90	-1.575	0.119
ZBI	55.29 ± 14.83	56.42 ± 12.83	-0.388	0.173

### Intervention effects

Repeated measures ANOVA was employed to evaluate intervention effectiveness. The repeated-measures ANOVA results are presented in **Table 2**. Since Mauchly’s sphericity test indicated that all four outcome measures violated the null hypothesis (*p* < 0.05), Greenhouse-Geisser correction was applied to the ANOVA results. Findings revealed a significant main effect of group on depressive outcomes (F = 16.6*25, df* = 1,*p* < 0.001), and a significant interaction effect between group and time (F = 5.940, df=2, p=0.005), indicating that changes in depressive symptoms over time differed between groups. For anxiety symptoms, the main effect of group was significant (F = 17.303, *df* = 1, *p* < 0.001), whereas the group × time interaction was not significant (F = 1.659, *df* = 2, *p* = 0.199). Because baseline GAD-7 scores were numerically higher in the control group than in the intervention group (p = 0.055), the group main effect should be interpreted cautiously. The non-significant group × time interaction indicated that the pattern of anxiety change over time did not differ significantly between groups. These findings do not support an additional effect of the psychoeducational intervention on anxiety symptoms compared with routine care. The main effect of group on post-traumatic stress outcomes was significant (F = 22.500, *df=1, p* < 0.001), and the interaction effect was significant (F = 4.366, *df=2*, p=0.020), indicating that changes in post-traumatic stress symptoms over time differed between groups. The main effect of group on caregiver burden outcomes was significant (F = 5.862, *df=1*, p=0.018), and the interaction effect was significant (F = 6.677, *df=2*, p=0.002), indicating that caregiver burden changed differently over time between groups.

**Table 2:** Repeated measures ANOVA.

Outcome	Group	T0	T1	T2	Model		
		M ± SD	M ± SD	M ± SD	F_Group_	F_Time_	F_Group*Time_
PHQ-9	Intervention	21.02 ± 6.90	16.93 ± 5.20	14.07 ± 5.11	16.625^***^	23.258^***^	5.940^**^
	Control	22.87 ± 7.33	22.40 ± 7.28	20.40 ± 6.82			
GAD-7	Intervention	17.56 ± 5.74	13.04 ± 3.40	12.27 ± 4.57	17.303^***^	30.073^***^	1.659
	Control	20.18 ± 6.98	17.47 ± 5.89	16.84 ± 5.36			
PCL	Intervention	41.67 ± 19.53	33.51 ± 10.71	29.76 ± 7.37	22.500^***^	12.822^***^	4.366^*^
	Control	47.58 ± 15.90	46.38 ± 13.76	44.13 ± 12.67			
ZBI	Intervention	55.29 ± 14.83	50.04 ± 13.93	43.33 ± 9.40	5.862^*^	12.475^***^	6.677^**^
	Control	56.42 ± 12.83	54.16 ± 14.43	54.42 ± 12.63			

T0, Baseline; T1, Post-intervention; T2, Follow-up. *p < 0.05, **p < 0.01, ***p < 0.001.

**Figure 2:**
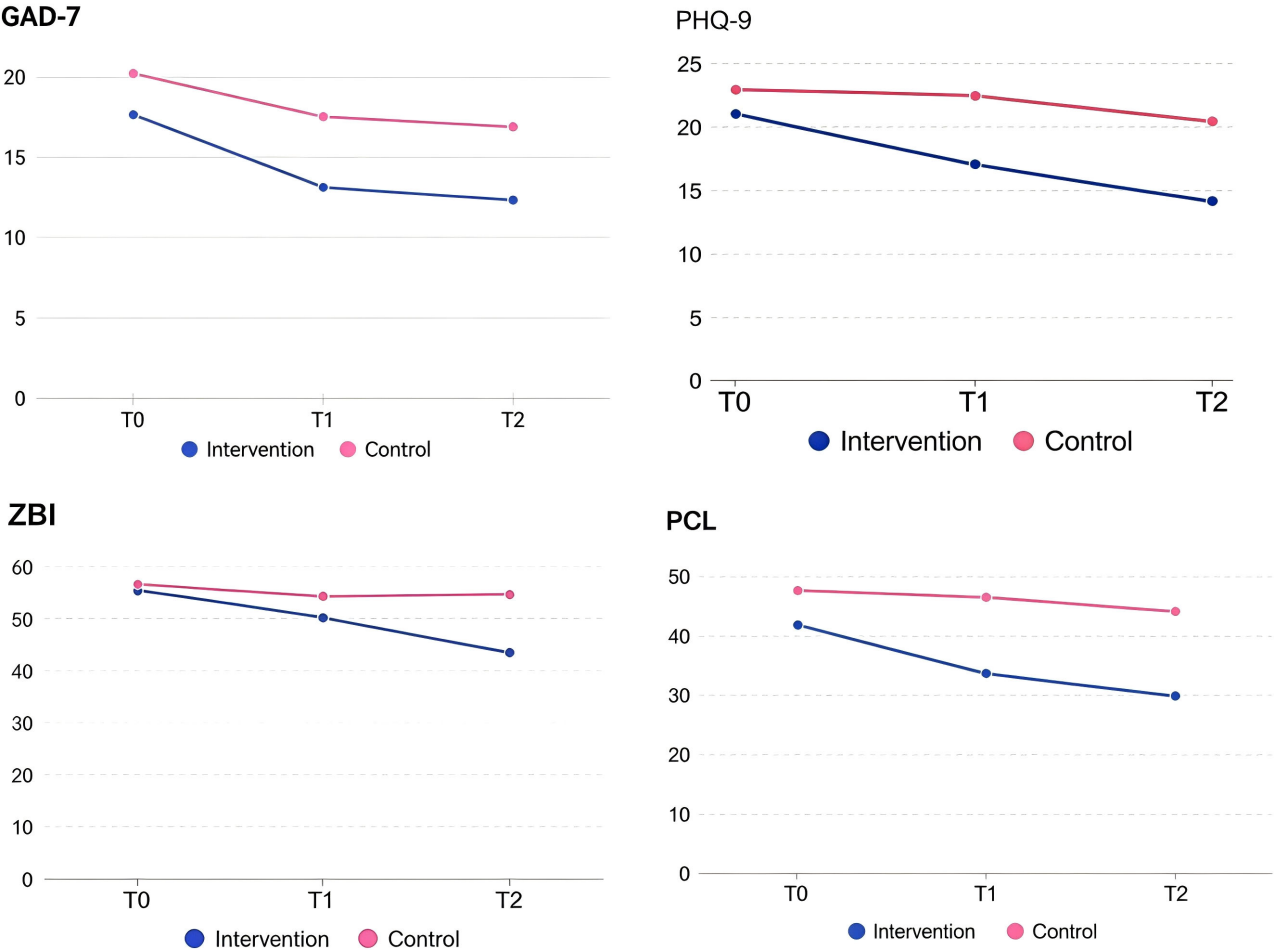
Trends in four outcome measures at three time points.

For indicators with significant interaction effects, *post hoc* tests were conducted to clarify changes within and between groups at different time points, and the results are presented in **Table 3**. Results indicated that for depressive symptoms, the experimental group showed significant reductions in depression scores at both post-intervention (T1) and follow-up (T2) compared to baseline (T0) (all *p* < 0.001). Furthermore, the T2 score was significantly lower than T1 (*p* = 0.020), demonstrating a sustained improvement trend. The control group exhibited no significant changes at any time point. For post-traumatic stress symptoms, the experimental group showed significantly lower symptom scores at both T1 and T2 compared to T0 (*p* < 0.01), while the control group exhibited no significant changes. Regarding caregiver burden, the experimental group showed a delayed improvement trend: no significant difference was observed between T1 and T0 (*p* = 0.057), but scores at T2 were significantly lower than both T0 and T1 (*p* < 0.05). The control group maintained stable burden levels across all time points. The longitudinal trends of the four outcome measures are shown in **Figure 2**.

**Table 3:** *Post-hoc* comparison results for significant interaction effects.

Outcome	Group	T0 vs. T1	T0 vs. T2	T1 vs. T2
PHQ-9	Intervention	-4.089***	-6.956***	-2.867*
	Control	-0.467	-2.467	-2.000
PCL	Intervention	-8.156**	-11.911***	-3.756
	Control	-1.200	-3.444	-2.244
ZBI	Intervention	-5.244	-11.956***	-6.711*
	Control	-2.267	-2.000	0.267

*p < 0.05, **p < 0.01, ***p < 0.001.

## Discussion

This study aimed to evaluate the effectiveness of a culturally adapted, structured psychoeducation intervention program developed for caregivers of pediatric hematologic/oncologic patients in China in alleviating depression, anxiety, post-traumatic stress symptoms, and caregiver burden. Caregivers receiving the intervention showed greater reductions in depressive symptoms and post-traumatic stress symptoms than those receiving routine care, and caregiver burden showed a delayed reduction at follow-up. However, the group × time interaction for anxiety symptoms was not significant, indicating that the intervention did not show an additional effect on anxiety beyond routine care.

### The effect of psychoeducation intervention

The observed reductions in depressive and post-traumatic stress symptoms are broadly consistent with previous psychoeducational and problem-solving intervention studies involving caregivers of children with cancer. For example, need-based psychoeducational intervention has been shown to reduce psychological distress among family caregivers of patients with leukemia (20), and problem-solving skills training for mothers of children newly diagnosed with cancer has demonstrated benefits for maternal adjustment and negative affectivity (9). Meta-analytic evidence among caregivers of children with cancer also suggests that psychoeducational interventions may improve post-traumatic stress symptoms, mood, and problem-solving skills, although effects are not consistently superior to usual care for all outcomes (8, 10). One possible interpretation is that the present intervention may have helped caregivers organize illness-related information, receive practical guidance, and develop more manageable ways of responding to the child’s treatment and emotional reactions. This explanation is consistent with prior psychoeducational and problem-solving approaches that emphasize information provision, coping skills, and structured problem solving (8–10). However, because the present study did not directly assess potential mediators such as perceived control, problem-solving ability, caregiving self-efficacy, or skill internalization, this explanation should be considered tentative.

This study also observed a delayed reduction in caregiver burden: no significant difference was observed immediately after the intervention, whereas caregiver burden was significantly lower at follow-up in the intervention group. This finding is consistent with evidence that caregiver intervention effects may unfold over time (21, 22). A possible explanation is that perceived caregiver burden is closely tied to ongoing caregiving tasks, family role adjustment, and confidence in managing practical problems. The knowledge and strategies provided in the intervention may therefore require time to be gradually internalized and applied in daily caregiving before changes in perceived burden become apparent. Nevertheless, this explanation should be interpreted cautiously because the present study did not directly measure changes in caregiving behavior, family role adjustment, or skill internalization.

The absence of a significant additional effect on anxiety should also be interpreted in context. Although anxiety scores decreased over time in both groups, the interaction was not significant. One possible explanation is that caregivers in both groups received routine financial resource consultation from medical social workers and disease-related education from healthcare providers after admission. These shared components of usual care may have contributed to the reduction in anxiety observed in both groups, thereby reducing the detectable difference between the intervention and control groups. This interpretation is consistent with broader evidence suggesting that social support, informational support, and psychoeducational interventions may be associated with reduced anxiety among cancer caregivers (23). However, the routine support components were not independently manipulated or quantified in the present study, so this explanation remains exploratory. In addition, as noted in the Results, baseline anxiety scores were numerically higher in the control group than in the intervention group, which should also be considered when interpreting the anxiety-related findings.

### Implications

This study has several theoretical and practical implications. Theoretically, it contributes to the emerging evidence base for caregiver-focused psychosocial support in pediatric hematology by examining a culturally adapted, manual-guided psychoeducational intervention for caregivers during the early post-diagnosis phase. The findings suggest that structured psychoeducation may be particularly relevant when intervention content is aligned with caregivers’ concrete communication dilemmas, family responsibilities, and support needs in the local clinical context. By incorporating formative qualitative work into intervention development, this study also illustrates a feasible pathway for translating culturally and clinically specific caregiver needs into structured psychosocial support.

Practically, the study suggests that a brief psychoeducational program delivered by trained medical social workers may be feasible within routine pediatric hematology services. Because caregivers in this setting already receive medical information, disease-related education, and social work support, a manual-guided intervention may serve as a structured supplement rather than a replacement for usual care. This model may help integrate psychological guidance, illness-related communication support, caregiver emotional regulation, and social resource navigation within existing hospital-based services.

## Limitations and future directions

This study has several limitations. First, the single-center design limits the representativeness of the sample and may affect the generalizability of the findings to other pediatric hematology settings. Second, the sample size was limited and the main analyses were based on a complete-case sample. Attrition may have introduced selection or survivorship bias, because caregivers who completed all assessments may have differed from those who withdrew in motivation, availability, distress level, or caregiving burden. Third, reductions in psychological symptom scores over time should not be attributed solely to the intervention. Caregivers may experience partial natural recovery or adjustment after the initial shock of diagnosis as they become more familiar with the treatment process, receive routine medical and social work support, and adapt to caregiving demands. In addition, statistical regression to the mean may have contributed to score reductions, particularly because caregivers showed high baseline levels of distress. Therefore, the observed changes should be interpreted cautiously. Fourth, the follow-up period was relatively short, limiting conclusions about the durability of intervention effects. Finally, the study relied on self-report measures and did not directly assess potential mechanisms such as perceived control, problem-solving ability, caregiving self-efficacy, family communication, or actual caregiving behavior.

Future studies should conduct multicenter randomized controlled trials with larger samples, longer follow-up periods, and analytic strategies that better accommodate missing data, such as mixed-effects models or multiple imputation. Future trials should also consider active or attention-matched control conditions and should directly assess potential mechanisms of change to clarify whether improvements are attributable to specific psychoeducational components, routine clinical support, natural recovery, or other contextual factors.

## Conclusion

This study provides preliminary evidence that a culturally adapted, manual-guided psychoeducational intervention may reduce depressive and post-traumatic stress symptoms among family caregivers of children newly diagnosed with hematologic malignancy. Caregiver burden did not show an immediate post-intervention reduction but decreased at follow-up, suggesting a delayed improvement pattern. In contrast, the intervention did not show an additional effect on anxiety symptoms compared with routine care. These findings support the potential value of integrating structured psychoeducational support into pediatric hematology settings, while also indicating that intervention effects may vary across psychological outcomes. Future multicenter studies with larger samples, longer follow-up periods, and more rigorous control conditions are needed to confirm the durability and mechanisms of these effects.

## Data Availability

The raw data supporting the conclusions of this article will be made available by the authors, without undue reservation.
